# Hepatic cytochrome P450 3A drug metabolism is reduced in cancer patients who have an acute-phase response

**DOI:** 10.1038/sj.bjc.6600448

**Published:** 2002-08-01

**Authors:** L P Rivory, K A Slaviero, S J Clarke

**Affiliations:** Medical Oncology, Level 6 Gloucester House, Sydney Cancer Centre, Missenden Road, Camperdown, NSW 2050, Australia; Department of Pharmacology, University of Sydney, 2006, NSW, Australia

**Keywords:** acute phase response, drug metabolism, erythromycin breath-test (EBT)

## Abstract

Inflammatory disease states (infection, arthritis) are associated with reduced drug oxidation by the cytochrome P450 3A system. Many chemotherapy agents are metabolised through this pathway, and disease may therefore influence inter-individual differences in drug pharmacokinetics. The purpose of this study was to assess cytochrome P450 3A function in patients with advanced cancer, and its relation to the acute-phase response. We evaluated hepatic cytochrome P450 3A function in 40 patients with advanced cancer using the erythromycin breath test. Both the traditional C_20min_ measure and the recently proposed 1/T_MAX_ values were estimated. The marker of acute-phase response, C-reactive protein and the pro-inflammatory cytokines IL-6, IL-1β, TNFα and IL-8 were measured in serum or plasma at baseline. Cancer patients with an acute phase response (C-reactive protein >10 mg l^−1^, *n*=26) had reduced metabolism as measured with the erythromycin breath test 1/T_MAX_ (Kruskal–Wallis Anova, *P*=0.0062) as compared to controls (C-reactive protein ⩽10 mg l^−1^, *n*=14)_._ Indeed, metabolism was significantly associated with C-reactive protein over the whole concentration range of this acute-phase marker (*r*=−0.64, Spearman Rank Correlation, *P*<0.00001). C-reactive protein serum levels were significantly correlated with those of IL-6 (Spearman coefficient=0.58, *P*<0.0003). The reduction in cytochrome P450 3A function with acute-phase reaction was independent of the tumour type and C-reactive protein elevation was associated with poor performance status. This indicates that the sub-group of cancer patients with significant acute-phase response have compromised drug metabolism, which may have implications for the safety of chemotherapy in this population.

*British Journal of Cancer* (2002) **87**, 277–280. doi:10.1038/sj.bjc.6600448 www.bjcancer.com

© 2002 Cancer Research UK

## 

Viral and bacterial infection, severe trauma and degenerative diseases are known to cause significant reductions in hepatic drug clearance, mostly through decreased expression of drug-metabolising cytochrome P450 enzymes (Morgan, 1997). This is mediated largely through down-regulation of gene transcription by the pro-inflammatory cytokines such as IL-6 and TNFα (Muntane-Relat *et al*, 1995; Morgan, 1997; Guillen *et al*, 1998; Pascussi *et al*, 2000). These cytokines also induce the synthesis of acute-phase reactants, such as C-reactive protein, by the liver (O'Riordain *et al*, 1999). This is usually accompanied by a decreased synthesis of albumin and pre-albumin. Most commonly, reduction in drug metabolism in the presence of acute-phase reactants involves the cytochrome P450 3A family (CYP3A), which is responsible for the metabolism of ∼60% of drugs used in medicine. This includes many of those used in cancer chemotherapy (e.g., taxanes, vinca alkaloids, camptothecins, tamoxifen, etoposide and oxazaphosphorines (Kivisto *et al*, 1995)).

The manner in which patients tolerate chemotherapy in general is remarkably unpredictable, and some experience significant morbidity leading to hospitalisation and, occasionally, mortality. Pro-inflammatory cytokines and acute-phase reactants are elevated in many patients with advanced cancer (Heys *et al*, 1998; Martin *et al*, 1999) and there is, therefore, the possibility that part of the inter-individual variability in drug clearance and toxicity could relate to the effects of these cytokines on CYP3A expression (Moreno *et al*, 1991; Craig *et al*, 1993; Chen *et al*, 1994; Muntane-Relat *et al*, 1995; Morgan, 1997; Pascussi *et al*, 2000). Further falls in CYP3A function may occur with age (Hunt *et al*, 1990) and reduced hepatic drug clearance may contribute to a greater risk of adverse events in elderly cancer patients (Yancik *et al*, 1998).

Pharmacokinetic variability in the disposition of anticancer drugs is responsible for a significant proportion of inter-individual variability in their activity and toxicity (Gurney, 1996). Surprisingly, the impact on drug metabolism of the acute-phase response that often accompanies cancer has not been explored in this setting. Hence, the purpose of the presented study was to estimate liver CYP3A function in patients with advanced cancer, to examine its association with the acute-phase response and to identify the key cytokines involved in the initiation of the latter. Serum levels of basic fibroblast growth factor (bFGF) and vascular endothelium growth factor (VEGF) have been shown to correlate strongly with tumour stage and outcome in several malignancies (Chen *et al*, 1999; Graeven *et al*, 1999; Ugurel *et al*, 2001). These may predict for more aggressive tumours. Hence, a secondary aim was to examine the relationship between the acute-phase response and circulating levels of bFGF and VEGF.

## METHODS

### Subjects

This was a prospective, single-centre study of the influence of acute-phase response on drug metabolism in cancer patients. It was open to all subjects >18 years of age with biopsy-proven, advanced malignancy who were about to receive chemotherapy. The entry criteria were: ECOG performance status (PS) 0-3, neutrophils >3.0×10^9^ l^−1^, bilirubin <2.0 ULN (upper limit of normal) and transaminases <2.5×ULN unless evidence of liver involvement (<5×ULN). The Ethics Committee of the Central Sydney Area Health Service approved the study, and written informed consent was obtained from all subjects.

### Experimental protocol

Blood samples were collected for routine evaluation of haematological and biochemistry parameters within 72 h prior to administering the EBT. Serum samples were also collected for the analysis of the acute-phase reactants C-reactive protein (CRP) and α_1_ acid glycoprotein (AAG) as well as albumin and pre-albumin and frozen at −70°C until analysis. The latter were performed by the Biochemistry Department of the Royal Prince Alfred Hospital using standard turbidimetric and nephelometry assays. Presence of an acute-phase response was defined as CRP >10 mg l^−1^.

The cytokines IL-1β, IL-6, TNFα and IFNγ were analysed in serum and IL-8, VEGF and bFGF in plasma using commercial ELISA kits (R&D, Minneapolis, MN, USA). Standard curves were run with each batch and only values greater than the lowest standard were reported (>15.6 pg ml^−1^ for IFNγ, TNFα, VEGF; >31.2 pg ml^−1^ for IL-8; >3.9 pg ml^−1^ for IL-1β, >1 pg ml^−1^ for bFGF and >3.13 pg ml^−1^ for IL-6).

The erythromycin breath test was performed as recently described (Rivory *et al*, 2000). Briefly, 4 μCi of ^14^C erythromycin (*N*-methyl-^14^C, 55 mCi mmole^−1^, NEN Life Science Products Inc, Boston, MA, USA) was injected intravenously and breath samples were collected into gas-tight balloons (Pytest®, Ballard Medical Products, Utah, USA) 5, 10, 15, 20, 25, 30 and 40 min later. These were processed by bubbling the collected gas through a capture solution consisting of hyamine hydroxide 10X (Packard, Sydney, NSW, Australia) in 50 : 50 methanol/ethanol v v^−1^ to which a trace of phenolphthalein was added. After the addition of scintillant (Ultima Gold®, Packard, Sydney, NSW, Australia) and counting, the data were expressed in terms of per cent of dose exhaled per minute at each time point by assuming a CO_2_ output of 5 mmoles min^−1^ m^−2^ (Watkins *et al*, 1989). The widely used measure of CYP3A activity, the flux at 20 min (C_20min_), was recorded (Hirth *et al*, 2000). In addition, the novel parameter, 1/T_MAX_, which correlates with total drug clearance of erythromycin (Rivory *et al*, 2000) was estimated from a fitting of a bi-exponential equation to the data as described recently (Rivory *et al*, 2000, 2001). In some cases, the profiles were extremely flat or had not reached a maximum at 40 min. In these cases, T_MAX_ was set at 50 min.

### Statistical analysis

The association between categorical (e.g. gender, ECOG) and continuous variables (e.g. EBT results, cytokine concentrations) was examined by Kruskal–Wallis Anova. Regression analyses between continuous variables were performed with the Spearman rank-order test.

The frequency distributions of CRP and AAG data were evaluated using the Kolmogorov-Smirnov One Sample Test. All tests were carried out using SYSTAT v 7.0.1 (SPSS Inc, Chicago, IL, USA) and *P*<0.05 was considered as significant.

## RESULTS

Between July 2000 and April 2001, a total of 40 subjects were investigated. These patients had mostly lung and breast cancer (see Table 1

**Table 1:**
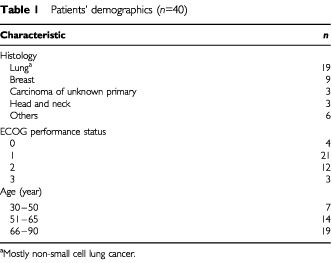
Patients' demographics (*n*=40)

) and ranged in age from 38 to 83, with a median of 64 years. There were similar numbers of males (*n*=21) and females (*n*=19).

The erythromycin breath test results were found to vary widely in this population. The median (and range in parentheses) for the C_20min_ measure was 0.050% min^−1^ (0.002–0.101) whereas it was 0.050 min^−1^ (0.02–0.12) for 1/T_MAX_. There was no significant effect of age or sex on either of the EBT parameters in this cancer population (Spearman Rank-Order and Kruskal–Wallis Anova, respectively).

Baseline serum CRP, AAG, albumin and pre-albumin were also variable and are summarised in Table 2

**Table 2:**
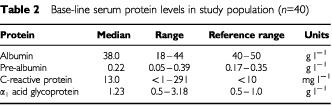
Base-line serum protein levels in study population (*n*=40)

. Only two patients had quantifiable serum TNFα (16.2, 18.5 pg ml^−1^), another two had quantifiable IFNγ (37.5, 77.9 pg ml^−1^), whereas most had quantifiable serum IL-1β (median: 10.6 pg ml^−1^). IL-6 was quantifiable in over half (*n*=33) with a median of 5.6 pg ml^−1^ (range: <3.2–193.5 pg ml^−1^). VEGF was found to range from <32.1 to 1537 pg ml^−1^ with a median of 274.6 pg ml^−1^. The range observed for bFGF was <1 pg ml^−1^ to 12.2 pg ml^−1^.

When the cancer patients were divided into control (⩽10 mg l^−1^) and acute-phase response (>10 mg l^−1^) groups based on the upper normal limit of serum CRP, those in the acute-phase group had an average 30% reduction in drug metabolism (0.070±0.024 vs 0.049±0.022 min^−1^, respectively). This was statistically significant (*P*=0.0062, Kruskal–Wallis Anova).

**Figure 1:**
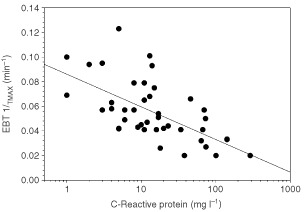
The relationship between the 1/T_MAX_ parameter of the erythromycin breath test and serum C-reactive protein in 40 patients with advanced cancer. The upper limit of normal of CRP is 10 mg l^−1^

**Figure 2:**
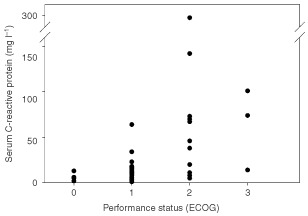
The distribution of the baseline serum C-reactive protein concentration in 40 cancer patients according to their performance status (ECOG). Analysis according to Kruskal–Wallis Anova test indicates significant differences between the four groups (*P*<0.006)

Further examination revealed that the effect occurred as a continuum with acute-phase response over the entire patient group. Indeed, the EBT 1/T_MAX_ values negatively correlated with both CRP and AAG with Spearman coefficients of −0.64 (*P*<0.00001) and −0.45 (*P*<0.005), respectively. Weaker correlations were observed against albumin and pre-albumin (data not shown). The EBT C_20min_ was only correlated with pre-albumin (Spearman coefficient=0.38, *P*<0.02). The distribution of the CRP values appeared to be log-normal and log-CRP was significantly correlated with 1/T_MAX_ as the independent variable (*r* ^2^=0.44, *P*<0.00002, Figure 1

). In comparison, a similar regression with the C_20min_ of the EBT yielded *r* ^2^=0.15 and *P*=0.012. CRP serum levels were significantly correlated with those of IL-6 (Spearman coefficient=0.58, *P*<0.0003) but not with any of the other cytokines. Also, the CRP levels were significantly different across the ECOG performance status categories (Kruskal–Wallis, *P*<0.006, Figure 2

). Because of the heterogeneous nature of the population in terms of disease site, the correlation between CRP and 1/T_MAX_ was also examined in the sub-groups of breast and lung cancer patients. The Spearman correlation values were −0.63 (*P*=0.07, *n*=9) and −0.53 (*P*<0.02, *n*=19), respectively, indicating that the effect is not likely to be tumour-type specific.

Co-medication may affect CYP3A activity either by induction or inhibition. Examination of the treatment files of the patients in this study revealed that three were being treated with inhibitors of CYP3A (diltiazem and clarithromycin, respectively), while four were on long-term treatment with the inducer dexamethasone (daily doses 2–4 mg). The mean±s.d. of 1/T_MAX_ for this latter group was 0.073±0.028 min^−1^ as compared to the study average of 0.057±0.024 min^−1^. The correlation between 1/T_MAX_ and CRP remained significant even after removal of the data from the seven patients on CYP3A-modifying medication (Spearman Rho=−0.55, *P*=0.002).

## DISCUSSION

These results indicate that CYP3A function in patients with advanced cancer is highly variable and correlates with markers of the acute-phase response. Those patients with an acute-phase response (CRP >10 mg l^−1^) had on average a 30% decrease in their metabolic activity as compared to the control group. This decrease in CYP3A activity with acute-phase response was best detected using the recent 1/T_MAX_ parameter of the erythromycin breath-test, which is a better predictor of total drug clearance (Rivory *et al*, 2001). However, similar trends occurred with the C_20min_ data, although these were not as significant. One of the disadvantages of using the C_20min_ approach is that values of this parameter are often significantly different between male and female subjects, possibly because of a flawed assumption regarding CO_2_ output (Rivory *et al*, 2001). This phenomenon, however, was not observed with our patient data. This suggests that the extreme variability in CYP3A metabolism observed in cancer patients obscures this possible bias.

The source of the variability in CYP3A function is not known but our observation of a significant correlation between acute-phase response and the EBT 1/T_MAX_ suggests that the pro-inflammatory cytokines, which are increased in malignancy (Heys *et al*, 1998; Barber *et al*, 1999; Martin *et al*, 1999), not only trigger the acute-phase response but also result in compromised drug metabolism by CYP3A in some cancer patients. The strong correlation between the IL-6 and CRP serum levels is in strong agreement with this interpretation, although other cytokines may have contributed. In fact, the biological effect of cytokines is modulated by complex inter-relationships with both their soluble and membrane-bound receptors. We argue that serum CRP, which is an indicator of hepatic gene regulation in the presence of inflammatory cytokines, reflects the overall biological effect of this inflammatory response.

AAG, which is one of the acute-phase reactants, was also increased and there is the possibility that the EBT was modified through the effects of protein-binding. Indeed, erythromycin is highly bound to this protein (Prandota *et al*, 1980). In our study, however, CRP was a more significant predictor of CYP3A activity than AAG. Also, it has been noted that the clearance of hepatically metabolised drugs is sometimes reduced in xenograft-bearing animals, even when these are not bound to AAG (Zamboni *et al*, 1998). Finally, there is evidence in support of a direct effect of pro-inflammatory cytokines on CYP3A expression, activity and drug clearance (Moreno *et al*, 1991; Craig *et al*, 1993; Chen *et al*, 1994; Muntane-Relat *et al*, 1995; Morgan, 1997; Pascussi *et al*, 2000).

The implications of this observation are many and of direct relevance to the chemotherapy of cancer. Firstly, the variability of CYP3A drug metabolism in cancer patients may justify the need for doses to be ‘individualised’, using measures such as the EBT (Hirth *et al*, 2000). Second, we found an association between acute-phase response and poor performance status in concert with other studies (O'Gorman *et al*, 1999). Hence, the link between acute-phase response and impaired drug metabolism may partly explain the observation of increased toxicity of drugs in patients with poor performance status (Krikorian *et al*, 1978; Freyer *et al*, 2000) although this is very likely a multi-factorial phenomenon.
